# Rapid Molecular Assays for the Detection of Yellow Fever Virus in Low-Resource Settings

**DOI:** 10.1371/journal.pntd.0002730

**Published:** 2014-03-06

**Authors:** Camille Escadafal, Oumar Faye, Amadou Alpha Sall, Ousmane Faye, Manfred Weidmann, Oliver Strohmeier, Felix von Stetten, Josef Drexler, Michael Eberhard, Matthias Niedrig, Pranav Patel

**Affiliations:** 1 Centre for Biological Threats and Special Pathogens 1, Robert Koch Institute, Berlin, Germany; 2 Department of Arboviruses, Institute Pasteur of Dakar, Dakar, Senegal; 3 Department of Virology, University Medical Centre, Göttingen, Germany; 4 Institute of Aquaculture, University of Stirling, Stirling, United Kingdom; 5 Laboratory for MEMS Applications, Department of Microsystems Engineering – IMTEK, University of Freiburg, Freiburg, Germany; 6 HSG-IMIT – Institut für Mikro- und Informationstechnik, Freiburg, Germany; 7 QIAGEN Lake Constance GmbH, Stockach, Germany; Aix Marseille University, Institute of Research for Development, and EHESP School of Public Health, France

## Abstract

**Background:**

Yellow fever (YF) is an acute viral hemorrhagic disease transmitted by *Aedes* mosquitoes. The causative agent, the yellow fever virus (YFV), is found in tropical and subtropical areas of South America and Africa. Although a vaccine is available since the 1930s, YF still causes thousands of deaths and several outbreaks have recently occurred in Africa. Therefore, rapid and reliable diagnostic methods easy to perform in low-resources settings could have a major impact on early detection of outbreaks and implementation of appropriate response strategies such as vaccination and/or vector control.

**Methodology:**

The aim of this study was to develop a YFV nucleic acid detection method applicable in outbreak investigations and surveillance studies in low-resource and field settings. The method should be simple, robust, rapid and reliable. Therefore, we adopted an isothermal approach and developed a recombinase polymerase amplification (RPA) assay which can be performed with a small portable instrument and easy-to-use lyophilized reagents. The assay was developed in three different formats (real-time with or without microfluidic semi-automated system and lateral-flow assay) to evaluate their application for different purposes. Analytical specificity and sensitivity were evaluated with a wide panel of viruses and serial dilutions of YFV RNA. Mosquito pools and spiked human plasma samples were also tested for assay validation. Finally, real-time RPA in portable format was tested under field conditions in Senegal.

**Conclusion/Significance:**

The assay was able to detect 20 different YFV strains and demonstrated no cross-reactions with closely related viruses. The RPA assay proved to be a robust, portable method with a low detection limit (<21 genome equivalent copies per reaction) and rapid processing time (<20 min). Results from real-time RPA field testing were comparable to results obtained in the laboratory, thus confirming our method is suitable for YFV detection in low-resource settings.

## Author Summary

Despite the use of a safe and effective vaccine, yellow fever virus is still causing hundreds of thousands of infections and tens of thousands of deaths every year. The disease is widespread in South America and Africa where several outbreaks have occurred in the past years. As the disease is difficult to distinguish from other illnesses during its early stage, it is necessary to develop reliable, rapid and simple diagnostic methods to confirm YF cases to be able to respond effectively to outbreaks through vaccination and vector control. In this study, we describe the development a diagnostic method for YFV, using an isothermal technology called recombinase polymerase amplification which allows detection of the virus within 20 minutes, using a portable and easy-to-use device. The YFV RPA assay proved to be a specific and sensitive detection method during testing in the laboratory and under field conditions in Senegal.

## Introduction

Yellow fever (YF) has been one of the most feared diseases during the past centuries, its historical impact ranking next to plague and smallpox. Unfortunately, unlike smallpox, YF virus (YFV) cannot be eradicated as its transmission by mosquitoes includes a sylvatic cycle. Despite the use of an effective vaccine since the 1930s, the World Health Organization (WHO) estimates that the disease affects more than 200,000 persons causing 30,000 deaths per year [1]. YF remains an important public health problem for the populations of 44 countries, 33 in Africa and 11 in Central and South America, where altogether almost 900 million people are at risk. In recent years, the number of YF cases has increased [2], and there is great concern that the disease might be introduced into new areas [3]. Recently, severe outbreaks have occurred in regions of Africa that have long been free of the virus, such as Darfur in Sudan or South Omo in Ethiopia which experienced the worst YF outbreak in Africa in 20 years in 2012 [4].

YFV is the prototype of the genus Flavivirus (family Flaviviridae) which comprises more than 80 positive-sense, single-stranded RNA viruses, including other human pathogens such as dengue, West Nile virus, Usutu virus, Zika virus, Japanese encephalitis virus and Tick-borne encephalitis virus [5].

Diagnosis of YFV infection is very challenging as the early symptoms caused by YFV are not specific. Laboratory confirmation is therefore essential for the differential diagnosis of YF with leptospirosis, malaria, viral hepatitis and other hemorrhagic diseases. Laboratory testing is also challenged by the short duration of the YF viremia in humans, the low-level laboratory infrastructure in most endemic areas and cross-reactions when using serological methods which lack specificity [6]–[8].

Alternatively, molecular diagnostic methods represent essential tools for early diagnostics as they are able to detect infections during the viremic phase. Early detection of cases is crucial to provide efficient patient management, rapid outbreak response and emergency vaccination measures. For this reason, considerable efforts are made to develop accessible direct detection methods based on molecular detection which allow a rapid and highly sensitive detection of YFV. Several molecular methods for YFV detection based on polymerase chain reaction (PCR), such as real-time RT-PCR, have been established, but these methods require the use of complex instruments and well-equipped laboratories [9]–[13]. However, in the case of direct detection methods for YFV, it is essential to be able to provide a portable, simple and robust method suitable for low-resource settings and field diagnosis, especially for outbreak response. For this reason, new molecular methods based on isothermal amplification have been developed for YFV detection, such as real-time reverse-transcription loop-mediated isothermal amplification (RT-LAMP) [14] and helicase-dependent amplification assays (HDA) [15].

In this study, we describe the establishment of a reverse-transcriptase recombinase polymerase amplification (RPA) assay for YFV detection. During RPA reaction, YFV RNA is first transcripted to DNA by a reverse-transcriptase. Secondarily, a phage derivated recombinase forms a nucleoprotein complex with the oligonucleotide primers which is able to scan for homologous sequences in the DNA template. RPA reaction can be performed between 25 and 42°C since denaturation of the DNA template is not required. If the target is present, the oligonucleotides are extended by strand displacing polymerases [16]. Real-time signal detection of the amplification can be performed within 15 minutes by using TwistAmp™ Exo probes (TwistDx, Cambridge, UK) and a ESEQuant Tube Scanner (QIAGEN Lake Constance GmbH, Stockach, Germany), a small easy-to-use fluorescence detection system which can perform eight measurements simultaneously. In low-resource settings where no power supply is available, the Tube Scanner device can be powered by a car adaptor, a small rechargeable battery or a battery charged by solar panels [17]. The RPA assay can also be integrated into a semi-automated system, using a GeneSlice microfluidic cartridge (HSG-IMIT, Freiburg, Germany) installed in a “SONDE” player device. As an alternative to real-time measurement, RPA results may be visualized after amplification on lateral-flow stripes (LFS) by using a different probe, TwistAmp™ Nfo (TwistDx, Cambridge, UK), during the RPA reaction. The reaction system can be stabilized in a dried formulation transportable without a cold chain.

## Materials and Methods

### Viruses and mosquito pools

Virus strains used were provided by the Robert Koch Institute in Berlin, the Bernhard-Nocht-Institute in Hamburg in Germany, and the Pasteur Institute of Dakar in Senegal. All virus strains were derived from cell culture, inactivated and stabilized. YFV strains are listed in Table 1 and other viral strains in Table 2.

**Table 1:** Yellow fever viral strains used for analytical specificity testing.

Virus description	Accession No.	Origin	Date	Lineage	real-time RT-PCR	RT-RPA	
YFV virus strains					Ct values	real-time Tt [min]	LFS
ArD 24553	_	Senegal	1976	_	24,6	3,3	n.d.
ArD 408/78	_	Burkina Faso	1978	_	23,9	3,0	n.d.
HD 117294	JX898868	Senegal	1995	6	16,5	2,3	n.d.
ArD 114891	_	Senegal	1995	6	16,0	1,6	n.d.
ArD 99740	_	Senegal	1993	3	25,0	5,1	n.d.
ArD 114991	_	Senegal	1995	_	24,3	3,4	n.d.
HD 122030	_	Senegal	1996	6	19,4	2,4	n.d.
ArD 122522	_	Senegal	1996	6	21,3	3,3	n.d.
HA 016/97	_	Liberia	1997	_	20,0	1,6	n.d.
HD 47471	_	Mauritania	1987	_	28,5	5,9	n.d.
ArD D X	_	Senegal	2000	5	21,4	2,4	n.d.
Asibi	AY640589.1	Ghana	1927	_	20,6	3,2	pos
ArD 114896	JX898871	Senegal	1995	3	20,3	3,1	pos
ArD 156468	JX898876	Senegal	2001	4	16,8	2,4	pos
DakArAmt7	JX898869	Ivory Coast	1973	1	15,4	2,1	pos
ArD 121040	JX898870	Senegal	1996	6	16,4	2,3	pos
ArD 149214	JX898873	Senegal	2000	5	15,5	2,2	pos
Ivory C 1999	AY603338.1	Ivory Coast	1999	6	19,1	2,5	pos
Trinidad 79A 788379	AF094612.1	Brazil	1979	3	20,1	2,5	pos
17D RKI #142/94/1	Vaccine strain	RKI	_	_	20,0	2,1	pos

n.d.: not determined; pos: positive; neg: negative; Tt: time threshold.

**Table 2:** Viral strains other than YFV used for analytical specificity testing.

Virus family	Virus specie	Virus strain	Real-time RT-PCR		RT-RPA result
			reference	result (Ct)	real-time/LFS
***Flaviviridae other than***	Dengue virus serotype 1	VR344 (Thai 1958 strain)		15.9	neg
***YFV***	Dengue virus serotype 2	VR345 (TH-36 strain)	[28] ih	18.8	neg
	Dengue virus serotype 3	VR216 (H87 strain)		20.3	neg
	Dengue virus serotype 4	VR217 (H241 strain)		16.2	neg
	West Nile virus lineage 1	Israel	[29]	19.9	neg
	West Nile virus lineage 2	Uganda		26.8	neg
	TB Encephalitis virus	K23 strain	[30]	16.3	neg
	RSS Encephalitis virus	Far eastern subtype		24.2	neg
	Japanese Encephalitis virus	ATCC SA14-14-2	[31]	19.4	neg
***Bunyaviridae***	Rift Valley Fever virus	strain ZH548	[32]	26.2	neg
***Filoviridae***	Ebola virus	Zaire strain	[33]	24.7	neg
	Marburg virus	Musoke strain		24.4	neg
***Alphaviridae***	Chikungunya virus	African isolate	ih	17.5	neg

TB: Tick-borne; RSS: Russian Spring Summer; neg: negative; ih: in-house assay.

Pools of mosquitoes, some of them infected with YFV, were provided by the Pasteur Institute of Dakar. The mosquito sampling protocol was extensively described by Diallo and colleagues [18].

### RNA extraction and sample preparation

Viral RNA was isolated from 140-µl aliquots of cell culture supernatants or 100-µl aliquots of mosquito pools, using the QIAamp Viral Mini Kit (QIAGEN Lake Constance GmbH, Stockach, Germany) according to the manufacturer's instructions. RNA was eluted in 100 µl of elution buffer and stored at −80°C until further use.

In order to use an energy-free method in the field trial, RNA extraction was performed with the innuPREP MP Basic Kit A (Jena Analytik, Jena, Germany) with a magnetic bead separation rack combined with proteinase K treatment according to the manufacturer's instructions. The nucleic acids were eluted in 100 µl of nuclease-free distilled water, and 5 µl were subjected to PCR or RPA, respectively.

RNA was extracted from 10-fold serial dilutions of YFV preparation and stored in aliquots at −80°C until use to assess the sensitivity of the extraction method. Human plasma samples spiked with low concentrations of YFV were used as a model for assay validation with clinical samples.

### Primer and probe design for RPA

All of the 79 YFV full-length sequences covering the 5′-UTR region available in the database (NCBI) were aligned using Geneious 5.0 software. According to Piepenburg and colleagues, primers of 30 nt to 35 nt in length are recommended for RPA [16]. One set of degenerate generic primers (YFV RF/RR) was designed according to the alignment for amplification of different YFV strains (Table 1). The primer sequences were identical for both lateral-flow strip RPA (LFS RT-RPA) and real-time RT-RPA primers, except for an additional biotinylation at the 5′ end of the LFS reverse primer. RPA exo probe for fluorogenic detection and RPA nfo probe for detection of dual-labeled amplicon were designed according to RPA guidelines from TwistDx (Cambridge, United Kingdom) and synthesized by TIB MOLBIOL (Berlin, Germany).

### Real-time RT-PCR

YFV-specific primer YFV FP/RP and probe YFV LNA2 were used to detect and quantify genomic RNA of YFV as described previously [9]. The assay was performed in a one-step format on the ABI 7500 instrument using the QuantiTect Virus Kit (QIAGEN Lake Constance GmbH, Stockach, Germany).

### Lateral-flow strip RT-RPA assay

LFS-RPA assay was performed using the TwistAmp™ nfo RT kit from TwistDx (Cambridge, United Kingdom) according to the manufacturer's instructions. Briefly, 29.5 µl of rehydration solution were mixed with 7.2 µl of PCR water, 2.1 µl of each primer (10 µM) and 0.6 µl of the target-specific RPA nfo probe (10 µM). Then 5 µl of RNA template was added to the 41.5 µl master mix. The template/master mix solution was added to the dry reagent pellet and mixed by pipetting up and down. Finally, the reaction was triggered by adding 3.5 µl of magnesium acetate (Mg(OAc)2, 280 mM) to the 46.5 µl reaction mix. The reaction mix was placed into the heating block at 39°C for 20 min, with brief mixing and centrifugation after 3–4 min of incubation. After amplification at 39°C for 20 min, 2 µl of amplification product was diluted in 100 µl of PBST buffer, and 10 µl of diluted amplicon was dropped on the sample pad of a HybriDetect lateral flow stripe (LFS) (Milenia Biotec, Giessen, Germany). Strips were then placed into tubes containing 100 µl of PBST buffer. The final result was read visually after 5 min of incubation. A test was considered positive when the detection line as well as the control line was visible. A test was considered negative when only the control line was visible.

### Real-time RT-RPA assay

Real-time RT-RPA assay was performed using the TwistAmp™ exo RT kit according to the manufacturer's instructions. The TwistAmp™ exo RT kit contains an additional RT-enzyme enabling the DNA amplification of RNA targets. Briefly, 37.7 µl of rehydration solution were mixed with 2.1 µl of each primer (10 µM) and 0.6 µl of the target-specific RPA exo probe (10 µM). Then 5 µl of RNA template was added to the 42.5 µl master mix. The template/master mix solution was added to the dry reagent pellet and mixed by pipetting up and down. Finally, the reaction was triggered by adding 3.5 µl of Mg(OAc)2 (280 mM) to the 47.5 µl reaction mix. The reaction tubes were mixed, centrifuged and then placed into the ESE Quant Tube Scanner for real-time monitoring of fluorescence. Reaction was performed at 39°C for 20 min, with brief mixing and centrifugation of reaction tubes after 3–4 min of incubation. This reaction temperature was determined optimal in terms of sensitivity. For data analysis, the Tube Scanner requires to be connected to a computer installed with the ESEQuant Tube Scanner software Version 1.0. Threshold values were determined by slope validation, i.e. slope (mV/min) values were compared in order to distinguish positive results from negative results. Further development and standardization of the method would allow using the device on its own with a direct display of positive or negative results for each sample.

### Analytical specificity of real-time and LFS RT-RPA

To test whether our assay is able to detect a wide variety of YFV strains, we utilized a panel of 20 different YFV strains described in Table 1. The analytical specificity was tested with a panel of 13 arboviruses and hemorrhagic fever viruses of which 9 are flaviviruses genetically related to YFV (Table 2).

### Analytical sensitivity of real-time and LFS RT-RPA

RT-RPA analytical sensitivity was evaluated by testing RNA extracts from 10-fold serial dilutions of YFV preparations comparatively to real-time RT-PCR used as the reference method. RNA was extracted from the YFV Asibi strain and RNA concentrations ranged from 2×10^5^ to 8 genome equivalent copies per reaction (GC/rxn). Repeatability of the method was assessed by testing each dilution 10 times with real-time RT-RPA and 5 times with LFS-RT-RPA.

### Centrifugal microfluidic cartridge

**Figure 1:**
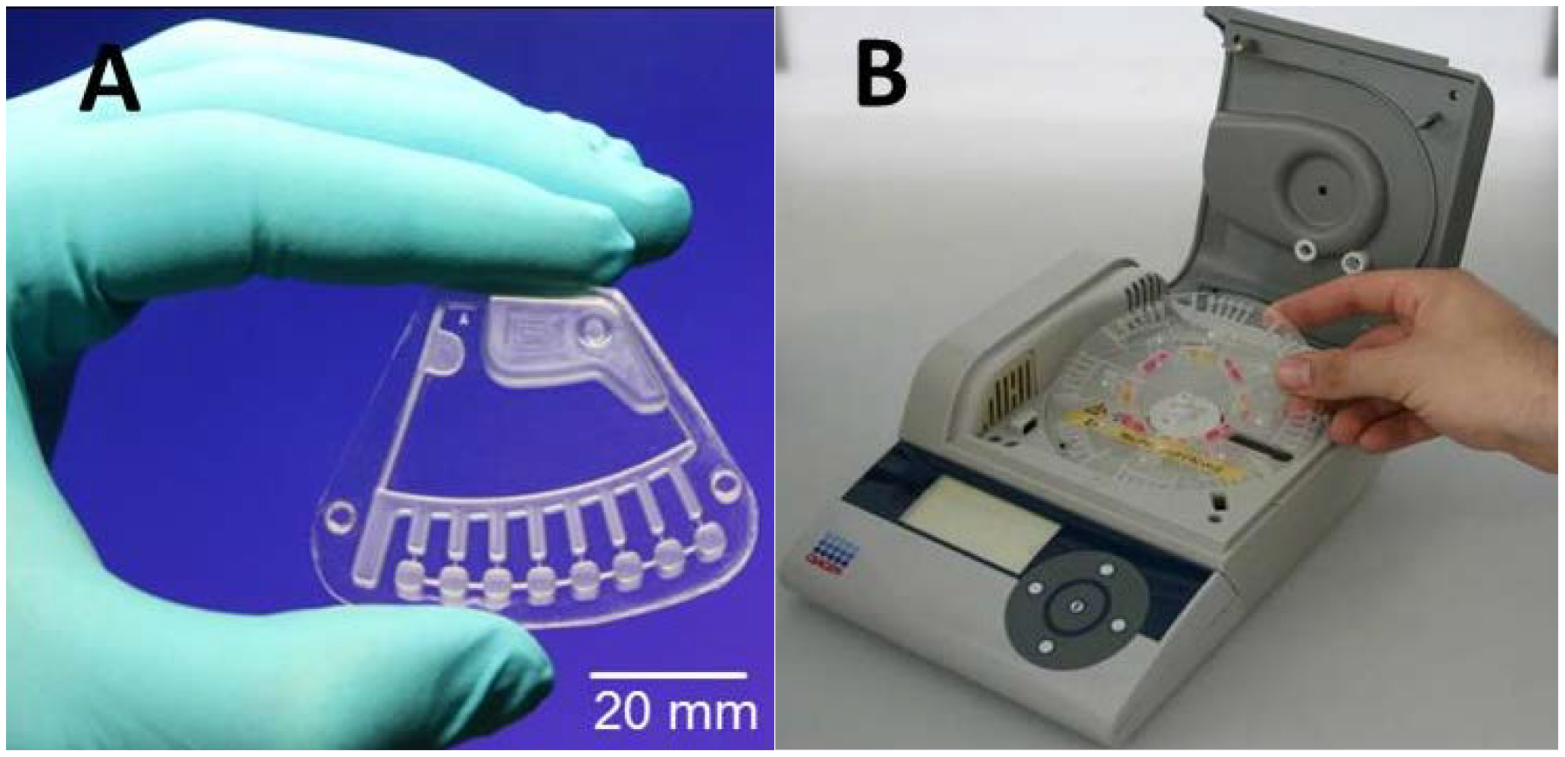
Centrifugal microfluidic platform. **A:** GeneSlice cartridge contains the microfluidic structure for aliquoting the reaction mix into eight 10 µl subvolumes; **B:** Prototype device for processing the GeneSlices (“SONDE player”) featuring defined rotation, acceleration and deceleration, heating and fluorescence detection (QIAGEN Lake Constance GmbH, Stockach, Germany).

Centrifugal microfluidic cartridges [19], termed GeneSlice (HSG-IMIT, Lab on a Chip Design- and Foundry Service, Freiburg, Germany), were used to demonstrate process automation of real-time RT-RPA in a small and portable processing device, the “SONDE” player, that may be used in the field with minimum manual interaction (Fig. 1B). The GeneSlice contains a microfluidic channel network that allows to aliquot an initial reaction mixture into 8 subvolumes by applying centrifugal forces. Each subvolume is then transferred into a separated amplification chamber (Fig. 1A) [20], [21].

The reaction mixture was composed of 73 µl rehydration solution, 4.2 µl forward/reverse primer (10 µM each), 1.2 µl probe (10 µM), 7 µl of Mg(OAc)2 (280 mM) and 10 µl of the DNA/RNA template. Three lyophilized pellets from the TwistAmp™ exo RT kit were resuspended in the 90 µl-reaction mixture. The reaction mixture is aliquoted into eight 10 µl volumes and transferred into amplification chamber by centrifuge force. Excess mixture is collected into a waste chamber. The “SONDE” player heats the samples at 41°C and RPA reaction is initiated in each amplification chamber. The fluorescence signal produced by the amplification is monitored for 20 minutes by the integrated detection unit. Amplification results were analyzed using IsoAmp Software (QIAGEN Lake Constance GmbH, Stockach, Germany).

### Field trial of real-time RT-RPA

Real-time RT-RPA assay combined with a magnetic bead-based extraction method was tested under field conditions in Senegal. Inactivated YFV virus and YFV RNA controls were prepared in dry-stabilized format using DNAstable Blood and RNAstable reagents (Biomatrica, San Diego, USA), respectively. These controls were stored at ambient temperature until further use in the field.

For the field trial of real-time RT-RPA, all reagents and instruments required were packed and transported by car from Dakar (14°43′12″N 17°28′48″W) to Mbour (14°25′19″N 16°57′51″W) at ambient temperature. At the Mbour city health center, the RPA setup was deployed and RNA was extracted from dry-stabilized virus controls using innuPrep MP basic kit. Subsequently, the extracted RNAs were tested with real-time RT-RPA for YFV. In order to reproduce field conditions where no power supply is available, the Tube Scanner was powered by a battery charged by solar panels.

## Results

### Primer and probe design for RPA

By analyzing the alignment of all available full genome sequences of YFV, the conserved 5′-non-coding region (NCR) of the YFV genome was chosen for primer and probe design (Table 3). The primer set YFV RF/RR efficiently amplified YFV RNA in LFS RT-RPA and real-time RT-RPA assays.

**Table 3:** List of primers and probe for the lateral-flow stripe and real-time RPA assay based on the YFV strain accession n° NC00203.

Assay format	Oligo name	Sequence 5′→3′	Direction	Position
**Lateral-flow stripe RT-RPA**	**YFV RF**	AAATCCTGTGTGCTAATTGAGGTGYATTGG	sense	4 to 33
	**YFV RR-Bio**	**Biotin-** ACATDWTCTGGTCARTTCTCTGCTAATCGC	antisense	93 to 122
	**YFV Rprobe nfo**	**FAM-** CTGCAAATCGAGTTGCTAGGCAATAAACAC **[THF]** TTTGGATT-AATTTTRATCGTT **-Ph**	sense	35 to 86
**Real-time RT-RPA**	**YFV RF**	AAATCCTGKGTGCTAATTGAGGTGYATTGG	sense	4 to 33
	**YFV RR**	ACATDWTCTGGTCARTTCTCTGCTAATCGC	antisense	93 to 122
	**YFV Rprobe exo**	gCAAATCgAgTTgCTAggCAATAAACACATT **[BHQdT]g[THF]A[FAMdT]** TAATTTTRATCgTTC **-Ph**	sense	37 to 87

**FAM**: 6-Carboxyfluorescein; **THF**: tetrahydrofuran; **Ph**: 3′phosphate to block elongation; **BHQ**: black hole quencher.

### Analytical specificity of real-time and LFS RT-RPA

The analytical specificity testing revealed that all the 20 different YFV strains were detected by both LFS and real-time RT-RPA assays. The testing results of the panel of 13 viruses other than YFV showed no cross-reactions, as all results were negative for both assays (Table 2). However, concerns with specificity were encountered with the LFS RT-RPA assay, as a faint band was observed in the negative controls when running time exceeded 5 minutes, thus potentially generating false-positive results.

### Analytical sensitivity of real-time and LFS RT-RPA

**Figure 2:**
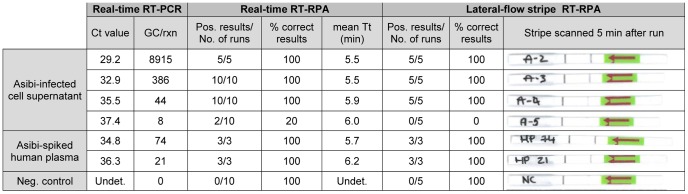
Sensitivity testing of the real-time and LFS RT-RPA with YFV cell supernatant and human plasma spiked with YFV in comparison with real-time RT-PCR results. Ct: cycle threshold; Tt: time threshold; Neg.: Negative; Pos.: Positive; Undet.: Undetermined.

**Figure 3:**
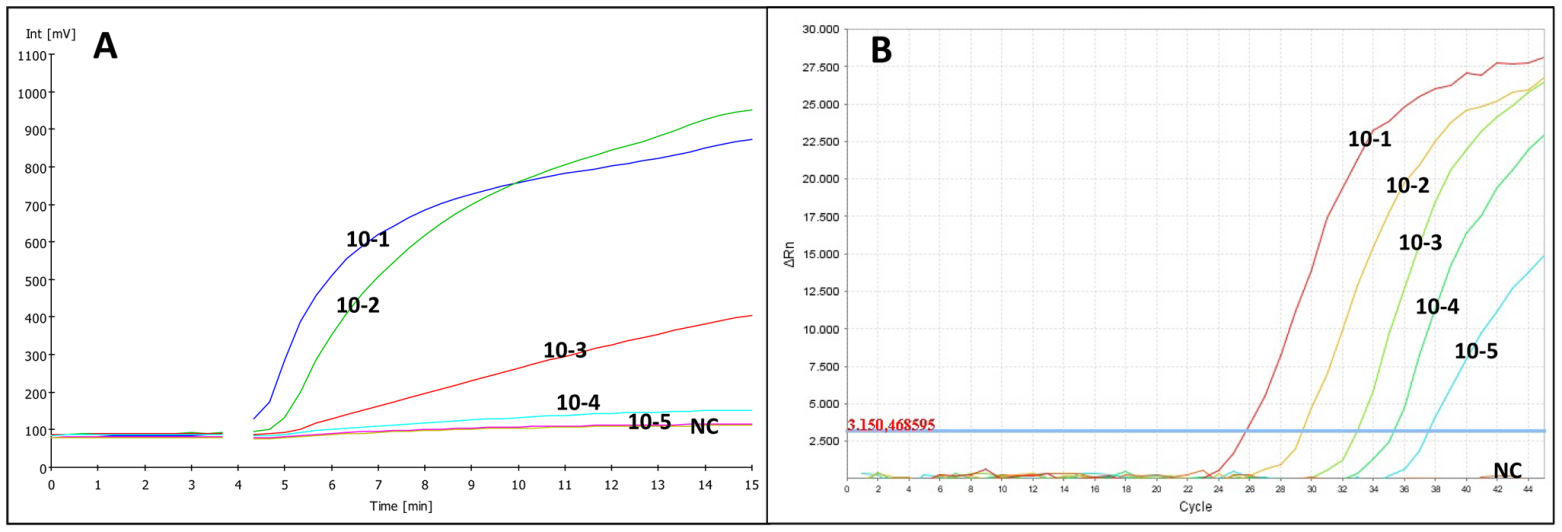
Amplification plots of real-time measurements for extracted RNA from 10-fold serial dilutions of YFV; A: RT-PCR results; B: RT-RPA results.

The analytical sensitivity of RT-RPA assays was evaluated by testing the RNA extracts from 10-fold serial dilutions of YFV preparations and by comparing real-time RT-RPA and real-time RT-PCR test results. The real-time RT-PCR showed linear results for the quantification of RNA standards over a range of 10 to 10^6^ genome copies. Real-time RT-PCR detected as low as 8 GC/rxn while real-time and LFS RT-RPA assays could detect as low as 44 GC/rxn in YFV RNA extracts and 21 GC/rxn for the testing of YFV-spiked human plasma samples (Figure 2). The amplification curves of the YFV RNA extracts from 10-fold serial dilutions are shown in Figure 3-A for real-time RT-RPA results and Figure 3-B for real-time RT-PCR results.

### Testing of mosquito pools with real-time RT-RPA on the Tube Scanner

Thirty-four samples of monospecific pools of wild-caught mosquitoes collected from Kedougou, southern Senegal were included in this study. The RNA extracts from these samples were tested in parallel with real-time RT-PCR and RT-RPA. Fourteen mosquito samples out of 34 (41.2%) resulted negative in real-time RT-PCR and 20 were positive (58.8%) with Ct values ranging from 24.65 to 35.51 (data not shown). Of the 20 samples detected positive in real-time RT-PCR, 16 were tested positive by real-time RT-RPA assay, providing a sensitivity of 80% (95% CI: 56.3% to 94.1%). Of the 14 samples tested negative in real-time RT-PCR, all were also tested negative by real-time RT-RPA assay, providing a specificity of 100% (95% CI: 76.7% to 100%). The overall agreement between the two assays was 88.4% (30/34) (Table 4).

**Table 4:** Performance of the real-time RT-RPA assay using the Tube Scanner or the GeneSlice cartridge in comparison to the reference method, real-time RT-PCR, for detecting YFV in mosquito pools.

		Real-time RT-PCR		Performance characteristics (%)			
		Positive	Negative	Sensitivity	Specificity	PPV	NPV
**RT-RPA on Tubescanner**	Positive	16	0	80%	100%	100%	77.8%
	Negative	4	14				
	Total (n = 34)	20	14				
**RT-RPA on**	Positive	10	0	71.4%	100%	100%	76.5%
**GeneSlice**	Negative	4	13				
	Total (n = 27)	14	13				

PPV: positive predictive value; NPV: negative predictive value.

### Testing of mosquito pools with real-time RT-RPA on the microfluidic platform

Twenty-seven RNA samples of mosquito pools were included in this part of the study. Thirteen mosquito samples out of 27 (48.1%) had negative results in real-time RT-PCR and 14 were positive (51.9%), with Ct values ranging from 27 to 35.5 (data not shown). Of the 14 samples tested positive with real-time RT-PCR, 10 were tested positive by real-time RT-RPA, providing a sensitivity of 71.4% (95%CI: 41.9% to 91.4%). All of the 13 samples that tested negative in real-time RT-PCR were also tested negative by real-time RT-RPA assay, providing a specificity of 100% (95% CI: 75% to 100%). The overall agreement between the two assays was 85.2% (23/27) (Table 4).

### Field testing of real-time RT-RPA

The virus and RNA controls were stabilized with DNAstable Blood and RNAstable reagents and tested in the laboratory using real-time RT-PCR and real-time RT-RPA. These results were compared to the testing results of the same amount of control samples without stabilizer, stored at −20°C. Results were comparable and proved the stabilization process to be effective. The average cycle threshold (Ct) and time threshold (Tt) values for all samples were 31.08 (SD = 0.74) and 5.5 (SD = 0.22), respectively. When stabilized controls and non-stabilized controls at −20°C were tested on real-time RPA during the field trial, the mean of Tt values of these samples was 5.3. These results are comparable to the values detected previously in the laboratory, indicating good reproducibility of the complete experimental workflow in the field.

## Discussion

In this study, we describe the development of a RT-RPA assay for YFV detection which can be performed without complex equipment in a basic laboratory setting, a rural health care center or an outbreak field investigation. We designed a set of primers and probe and developed a real-time methodology which enables to detect down to 21 GC/rxn. This detection limit is slightly higher than the 8 GC/rxn detected by real-time PCR [9]. Nonetheless, this level of sensitivity is sufficient to detect wild-type YFV in natural infections or serious adverse events (SAEs) following YFV immunization which produce viremia levels up to 10^8^ PFU/ml [22]–[24].

Test results for spiked human plasma samples indicated that serum does not affect significantly the assay sensitivity. Therefore, we can assume that the test can be applied for laboratory case confirmation of suspected YFV cases. However, there is further need to validate intensively the assay using YF clinical samples from various endemic countries and from patients at different stages of the disease.

The LFS-RPA assay experienced specificity problems, as a faint nonspecific band appeared in the negative controls when running time exceeded 5 minutes. Such faint bands have not been observed neither for the very low dilutions of YFV RNA nor during testing of other viruses. Therefore, these false-positive results are not due to contamination but rather to the clotting of proteins or primers which could not bind to any template. Unequivocal interpretation of LFS may be provided by an ESEQuant Lateral Flow Reader (QIAGEN Lake Constance GmbH, Stockach, Germany). However, at this point, we recommend particular caution during LFS operation and interpretation and further optimization of the assay before use under field conditions.

Real-time RT-RPA results demonstrated an optimal specificity. Testing results of the mosquito pools demonstrated an analytical specificity of 100% on both the Tube Scanner and the microfluidic GeneSlice cartridge. Real-time RT-RPA on the microfluidic GeneSlice cartridge showed a statistically similar sensitivity (71.4% and 80% respectively) as the confidence intervals of both sensitivity values overlap. The lower sensitivity value of the GeneSlice method might be due to the complexity of the microfluidic unit operations which comprises release of liquid reagents, reconstitution of lyophilized reagents, aliquoting the sample into eight independent reaction cavities and mixing of reagents with the RNA samples. Nevertheless, the performance of the GeneSlice is satisfying, and no cross-contamination between wells was observed. Moreover, this semi-automated and downscaled system leads to a significant reduction in costs, manual work and waste, making it an attractive method for point-of-care applications such as the screening for hemorrhagic fevers in Africa. However, the real-time RT-RPA on the Tube Scanner was used for further evaluation including in field conditions because of its higher sensitivity.

During the field study, real-time RT-RPA has demonstrated similar performance to that during previous testing under laboratory conditions. Based on our results, the assay proves to have great potential as a point-of-care molecular diagnostic method for various reasons: all reagents are lyophilized with the main RPA reagents provided in a single dried pellet, which simplifies assay preparation and allows long-term storage at room temperature; amplification is performed at constant temperature; ESEQuant Tube Scanner device is significantly lighter, smaller and cheaper than all other available mobile PCR cyclers or turbidimeter devices for LAMP assays; the assay has a low energy consumption; reaction times are short and the system is simple, robust and portable.

The cost is approximately 4 euros per test for real-time RPA and 5 euros per test for real-time RT-PCR in lyophilized form. At this stage, costs per sample for both techniques are comparable. However RT-RPA is a newly developed technique and prices are likely to decrease in the future while availability and throughput will increase. Furthermore, the detection device for real-time RPA is approximately 10 times cheaper than a real-time PCR machine.

An external quality assessment study on diagnostic methods for YFV infections launched in 2011 revealed that the main weakness observed for molecular methods was the inability of some assays to detect the YFV genome of wild-type strains, whereas the vaccine strain was always detected [25]. This specificity problem has not been observed for the YFV RT-RPA assay, as all YFV strains were detected. Furthermore, our assay revealed no cross-reactions with other closely related viruses.

Recently, another isothermal amplification method for YFV detection was developed based on reverse transcription loop-mediated isothermal amplification (RT-LAMP) technology [14]. In contrast to RPA, LAMP requires a larger set of six primers, a higher temperature (62°C) and a longer run time. Sensitivity is not comparable, as results of RT-LAMP were expressed as PFU instead of GC detected, but RT-LAMP usually presents equal or lower sensitivity than RPA [26], [27]. In fact LAMP uses nonspecific intercalating fluorophores for detection while RPA uses specific detection probes.

In summary, we have developed a very rapid and sensitive isothermal RPA assay in real-time and lateral-flow stripe format for the detection of YFV. Both of these assays can be easily applied in low-resource settings as an alternative to traditional laboratory-based molecular diagnostic assays. However, the LFS format needs further optimization to exclude all risks of false-positive results. The real-time RT-RPA assay, using the transportable Tube Scanner device combined with the RNA extraction method based on magnetic beads, and the use of lyophilized reagents which can be stored at ambient temperature allowed us to apply our RPA assay under field conditions in Senegal with performance similar to that of cutting-edge laboratory settings.
